# Lingual Lymph Node Metastases as a Prognostic Factor in Oral Squamous Cell Carcinoma—A Retrospective Multicenter Study

**DOI:** 10.3390/medicina57040374

**Published:** 2021-04-12

**Authors:** Masahiro Kikuchi, Hiroyuki Harada, Ryo Asato, Kiyomi Hamaguchi, Hisanobu Tamaki, Masanobu Mizuta, Ryusuke Hori, Tsuyoshi Kojima, Keigo Honda, Takashi Tsujimura, Yohei Kumabe, Kazuyuki Ichimaru, Yoshiharu Kitani, Koji Ushiro, Morimasa Kitamura, Shogo Shinohara, Koichi Omori

**Affiliations:** 1 Department of Otolaryngology—Head & Neck Surgery, Graduate School of Medicine, Kyoto University, Kyoto 606-8507, Japan; m_kitamura@ent.kuhp.kyoto-u.ac.jp (M.K.); omori@ent.kuhp.kyoto-u.ac.jp (K.O.); 2 Department of Otolaryngology—Head & Neck Surgery, Medical Research Institute, Kitano Hospital, Osaka 530-8480, Japan; h_harada@ent.kuhp.kyoto-u.ac.jp; 3 Department of Otolaryngology—Head & Neck Surgery, National Hospital Organization Kyoto Medical Center, Kyoto 612-8555, Japan; asato@ent.kuhp.kyoto-u.ac.jp; 4 Department of Otolaryngology—Head & Neck Surgery, Kobe City Medical Center General Hospital, Hyogo 650-0047, Japan; k_hamaguchi@ent.kuhp.kyoto-u.ac.jp (K.H.); sinosino@kcho.jp (S.S.); 5 Department of Otolaryngology—Head & Neck Surgery, Kurashiki Central Hospital, Okayama 710-8602, Japan; orl-tamaki@nifty.com (H.T.); m_mizuta@ent.kuhp.kyoto-u.ac.jp (M.M.); 6 Department of Otolaryngology, Tenri Hospital, Nara 632-0015, Japan; entrh2000@gmail.com (R.H.); t_kojima@ent.kuhp.kyoto-u.ac.jp (T.K.); 7 Department of Otolaryngology, Japanese Red Cross Wakayama Medical Center, Wakayama 640-8558, Japan; kegohonda@gmail.com; 8 Department of Otolaryngology, Head and Neck Surgery, Japanese Red Cross Otsu Hospital, Shiga 520-0046, Japan; ttakashi502@gmail.com; 9 Department of Otolaryngology-Head & Neck Surgery, Hyogo Prefectural Amagasaki General Medical Center, Hyogo 660-8550, Japan; pa45328@gc4.so-net.ne.jp; 10 Department of Otolaryngology—Head & Neck Surgery, Kokura Memorial Hospital, Fukuoka 802-8555, Japan; ichimaru-k@kokurakinen.or.jp; 11 Department of Otorhinolaryngology—Head & Neck Surgery, Shizuoka General Hospital, Shizuoka 420-8527, Japan; y_kitani@ent.kuhp.kyoto-u.ac.jp; 12 Department of Otorhinolaryngology—Head & Neck Surgery, Shiga General Hospital, Shiga 524-8524, Japan; koji.ushiro@gmail.com

**Keywords:** lingual lymph node, metastasis, squamous cell carcinoma, oral carcinoma, lingual tumor

## Abstract

*Backgrounds and Objectives:* The epidemiology and prognostic role of lingual lymph node (LLN) metastasis in patients with oral squamous cell carcinoma (OSCC) remain unclear. Here, we aimed to analyze the clinicopathological features, risk factors, and prognostic role of LLN metastasis in patients with OSCC. *Materials and Methods:* In total, 945 patients with OSCC were retrospectively analyzed. Clinicopathological features were compared between patients with and without LLN metastasis. The risk factors of LLN metastasis and its effects on survival outcomes were evaluated using multi-variate analysis. *Results:* LLN metastasis was noted in 67 patients (7.1%). Habitual alcohol consumption and clinical neck node metastasis were independent risk factors for LLN metastasis. LLN metastasis was an independent prognostic factor for disease-free and overall survival, although LLN dissection did not improve survival outcomes. *Conclusion:* LLN metastasis is an independent adverse prognostic factor. Further prospective studies are needed to fully assess the extent of LLN dissection required in OSCC patients.

## 1. Introduction

Lymph of the anterior oral cavity is drained primarily into the level I lymph nodes. In contrast, drainage from the lateral oral tongue and posterior floor of the mouth is directed toward level II lymph nodes [1]. Apart from these, there are para-lingual intermediate lymph nodes, known as “lingual lymph nodes (LLNs)”, located between the lingual structure and cervical lymph nodes [2]. Although oral cancer can metastasize to LLNs and serve as the starting point of local recurrences [3], little attention has been paid to these nodes because of the low incidence of LLN metastasis. In 1985, Ozeki et al. [2] reported three cases of tongue cancer with LLN metastasis within the lingual musculature. Since then, this form of metastasis has received more attention from clinicians because LLNs can be left behind after a standard (modified/radical) neck dissection owing to their location (beyond the area of dissection).

LLNs were first reported by Rouviere et al. [4], and they were categorized into two groups: median LLNs (MLLNs), located in the lingual septum, and lateral LLNs (LLLNs), located in the region lateral to the genioglossus, geniohyoid, or hyoglossus muscles. The definition of LLLN proposed by Rouviere did not clearly distinguish LLLNs from submandibular nodes (level IB); however, recent published articles defined LLLNs as lymph nodes located between the genioglossus and hyoglossus muscles, along the lingual artery [5,6]. Ando et al. reported a new group of LLNs called “para-hyoid nodes”, which are located in the area where the lingual artery originates from the external carotid artery and passes deep into the post-lateral border of the hyoglossus muscle behind the hypoglossal nerve [7,8]. Many authors have reported LLN metastasis in case reports or series [5,8,9,10,11,12,13], but the epidemiology and the prognostic role of LLN metastasis in patients with oral squamous cell carcinoma (OSCC) [14,15] remain unclear. Therefore, in this multi-center study, the clinicopathological features, risk factors, and prognostic role of LLN metastasis in patients with OSCC were analyzed.

## 2. Materials and Methods

### 2.1. Patient Population and Clinicopathological Features

This study was performed in 12 hospitals associated with Kyoto University and its Affiliated Hospitals—Head and Neck Oncology Group (Kyoto–HNOG) in Japan. Clinical data for each patient were retrospectively extracted from medical charts. OSCC patients who underwent definitive surgery with or without induction chemotherapy or postoperative radiotherapy (PORT) between March 2010 and February 2017 were included in this study. Indication for postoperative radiotherapy included positive margin, multiple cervical lymph node metastasis, and extra-capsular extension. Meanwhile, patients with distant metastasis and those who underwent definitive radiotherapy instead of surgery were excluded from the study. This study was approved by the institutional review board of each participating institution and was led by the Kyoto University Certificated Review Board (ethics code: R2201, date of approval: 9 October 2019). Informed consent was not required because of the retrospective nature of this study. However, regarding data use in this retrospective study, the patients were given the opportunity to opt out of the study at any time, which was announced on the website of each institution.

Data on age, sex, tumor subsite, smoking or drinking habits, oral hygiene, presence of diabetes mellitus, tumor-node-metastasis (TNM) classification, pretreatment neutrophil-to-lymphocyte ratio (NLR), induction chemotherapy, surgical method (primary resection, neck dissection, or LLN dissection), postoperative radiotherapy, resection margin of resected tumor, extracapsular extension of resected lymph nodes, and tumor differentiation were collected and analyzed. Habitual drinkers were defined as those who consumed at least one alcoholic drink per day, and current smokers were defined as those who smoked daily. Poor oral hygiene was defined as the presence of caries, and any dental plaque or food debris around the teeth. NLR was defined as the absolute neutrophil count divided by absolute lymphocyte count within two weeks before initial treatment [14].

### 2.2. Patients with LLN Metastasis

Previous reports [5,6,7] have defined two subcategories for LLNs. Median LLNs (MLLNs) are nodes located in the lingual septum, while lateral LLNs (LLLNs) are nodes which lie between the genioglossus or geniohyoid muscle medially and hyoglossus muscle laterally (classical LLLNs). Moreover, nodes located at the “para-hyoid” area, which includes the area where the lingual artery originates from the external carotid artery and passes deep to the post-lateral border of the hyoglossus muscle, hypoglossal nerve, and the cornu of the hyoid bone, were proposed by Ando et al. [7] to be classified under LLLNs.

**Figure 1:**
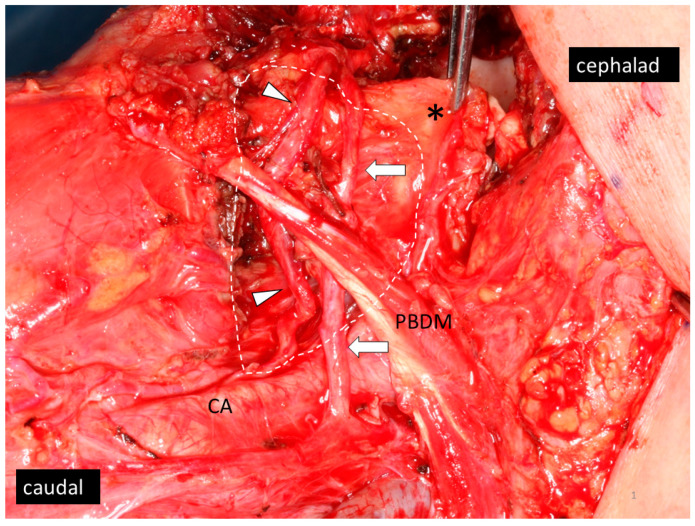
After the dissection of a lingual lymph node (LLN) in the left side of the neck. White dotted line shows the dissected area. In this case, the primary tumor was removed by the pull-through approach. Forceps are pinching the preserved base of tongue (asterisk). The hyoglossus muscle was partially removed. The lingual artery (arrow heads) and the hypoglossal nerve (arrows) were preserved. CA, carotid artery; PBDM, posterior belly of digastric muscle.

Therapeutic LLN dissection was performed for a patient with a clinically apparent LLN (a round-shaped LLN or LLN with higher ^18^F-fluoro-2-deoxy-d-glucose (FDG) uptake in positron emission tomography (PET) compared to background) was detected before surgery. In contrast, the need for prophylactic LLN dissection was decided per the surgeons’ preference. In cases where the primary tumor was resected transorally, the extent of LLN dissection was confined to the para-hyoid area, which includes LLN (if any), and the loose fibrofatty tissue along the lingual artery from the root of the artery toward the hyoglossus muscle. On the other hand, when the primary tumor was resected by the pull-through approach, the extent of LLN dissection extended from the para-hyoid area to the adipose tissue in the mouth floor and including the sublingual gland, even if an LLN was not detected. The hypoglossal nerve was preserved during the procedures (Figure 1).

**Figure 2:**
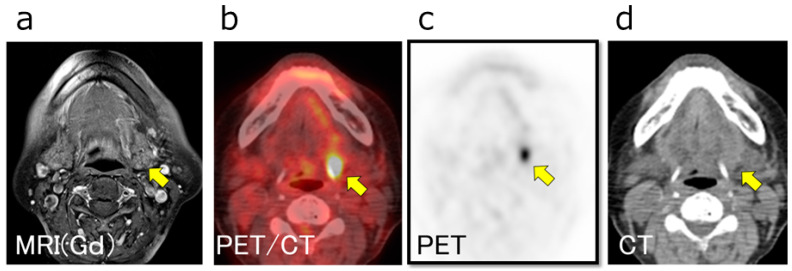
Representative case with LLN metastasis. A 79-year-old woman with a left tongue squamous cell carcinoma (SCC) (cT4aN2bM0) showed lateral lingual lymph node (LLLN) metastasis upon preoperative imaging studies. (**a**) Contrast-enhanced magnetic resonance imaging (MRI); (**b**) positron emission tomography–computed tomography (PET/CT); (**c**) PET; and (**d**) low-dose CT performed in conjunction with the PET scan. An LLLN on the left side (arrow) was not clearly detected by contrast-enhanced MRI, whereas it was clearly identified by PET/CT.

Furthermore, initially detected LLN metastasis (iLLN) was defined as histologically proven LLN at initial definitive surgery with therapeutic or prophylactic LLN dissection. Recurrent LLN metastasis (rLLN) was defined as LLN that was proven histologically positive or clinically apparent on imaging analysis but lacked local recurrence. In this study, LLN metastasis in the patients was of either iLLN or rLLN type. A representative patient with LLN metastasis is shown in Figure 2.

The clinicopathological features of patients with and without LLN metastasis were compared. Among the probable risk factors of clinicopathological features at the initial stage, significant risk factors for LLN metastasis were analyzed independently using the forced method in the multivariate logistic regression analysis. In addition, the effects of LLN on survival outcomes, particularly disease-free survival (DFS) and overall survival (OS), were evaluated.

### 2.3. Statistical Analysis

Fisher’s exact test was used to evaluate and compare clinicopathological findings between patients with and those without LLNs. The Mann–Whitney U test was used to compare NLR between patients with and those without LLN. To predict LLLN metastasis, multivariate analysis using a binary logistic regression test (forced method) was performed. Survival outcomes, including DFS and OS, were estimated using the Kaplan–Meier method, and groups were compared using the log-rank test. Cox proportional hazard regression models were used to determine the relationships between patient clinicopathological characteristics and DFS or OS outcomes. Multivariate Cox proportional hazard regression was performed using the forced entry method. Results with *p*-values less than 0.05 were considered statistically significant. SPSS software version 25 (SPSS Japan Inc., Tokyo, Japan) was used for statistical analysis.

## 3. Results

### 3.1. Patient Population and Clinicopathological Features

In total, 945 (580 male and 365 female) OSCC patients were enrolled in this study. The median age was 69 years (range 20–97 years). Tumor subsite distribution was as follows: lingual 57.1% (540 patients), gingiva 22.0% (208 patients), oral floor 10.5% (99 patients), buccal 8.4% (79 patients), hard palate 1.4% (13 patients), and lip 0.6% (6 patients). Eighty-six percent (813 patients) did not have tumors that extended beyond the midline, whereas the remaining 14.0% (132 patients) had tumors that extended beyond the midline. With regard to drinking and smoking habits, 45.6% (431 patients) drank alcohol and 26.0% smoked tobacco. A total of 66.8% (631 patients) had good hygiene, whereas 33.2% (314 patients) had poor hygiene. There were 138 patients (14.6%) with diabetes mellitus (DM). Clinical TNM classification in the 8th edition was T1/2/3/4 = 273 (28.9%)/346 (36.6%)/139 (14.7%)/187 (19.8%), N0/1/2/3 = 646 (68.4%)/117 (12.4%)/169 (17.9%)/13 (1.4%), and M0/1 = 945 (100%)/0 (0%). The median NLR was 2.3, with a range from 0.4 to 39.6.

Neoadjuvant chemotherapy was performed in 226 patients (23.9%). The primary tumor was resected using an exclusively transoral approach in 783 patients (82.9%), whereas a transcervical pull-through procedure was performed in the remaining 162 patients (17.1%). Neck dissection was simultaneously performed in 510 patients (54.0%). Among them, 271 patients (28.7%) underwent LLN dissection after usual neck dissection, including 46 patients who underwent therapeutic LLN dissection, and the remaining 225 patients who underwent prophylactic dissection. Post-operative radiotherapy with or without chemotherapy was performed in 145 patients (15.3%).

Histopathological examination after surgery revealed that the primary resection margin was negative in 870 patients (92.1%) and positive in 75 patients (7.9%). Extra capsular extension was seen in 62 patients (6.7%). A total of 892 patients (94.4%) had well or moderately differentiated tumors, and 53 patients (5.6%) had poorly differentiated tumors.

All surviving patients were followed up for a median of 60 months (range 2.6–152 months). At the last follow-up, 132 patients had died of OSCC and 77 had died of other causes. The five-year OS rate was 78.0% (95% CI: 77.97–78.03%). Local and regional recurrences recurred in 154 and 166 patients, respectively. On the other hand, distant metastases occurred in a total of 83 patients. The five-year DFS rate was 70.7% (95% CI: 70.67–70.73%).

### 3.2. Patients with LLN Metastasis

**Figure 3:**
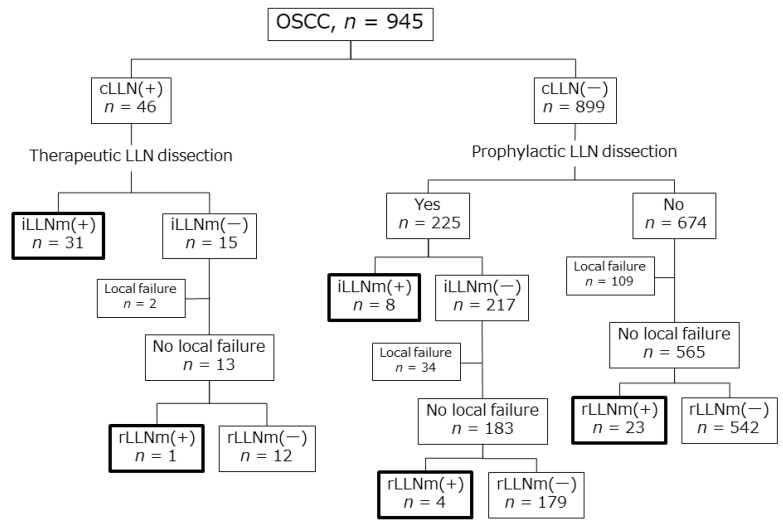
Patient population distribution. OSCC, oral squamous cell carcinoma; cLLN, clinically apparent lingual lymph node; iLLNm, initial lingual lymph node metastasis; rLLNm, recurrent lingual lymph node metastasis. LLN metastasis (iLLNm or rLLNm) was enclosed by a heavy line.

Before initial therapy, 46 of 945 patients (4.9%) were LLN (+) clinically, whereas 899 (95.1%) were LLN (−) clinically. Therapeutic LLN dissection was performed for the 46 clinically LLN (+) patients. Of the 46 LLN (+) patients, 31 (71.7%) were revealed to have LLN (+) pathologically. Among the 15 patients who were initially diagnosed as LLN (+) clinically but as LLN (−) pathologically after therapeutic LLN dissection, one patient showed LLN recurrence without local recurrence. Among the 899 clinically LLN (−) patients, 225 underwent prophylactic LLN dissection, and occult LLN metastasis was detected in eight patients (3.6%; 8 of 225). Four patients among the 217 patients who were first diagnosed as LLN (−), both clinically and pathologically, showed LLN recurrence without local recurrence after prophylactic LLN dissection. Of the remaining 674 clinically LLN (−) patients who did not undergo prophylactic LLN dissection, 23 showed LLN recurrence without local recurrence. In total, LLN metastasis was observed in 67 patients and had an incidence rate of 7.1% (67 of 945). Among them, 39 were initially detected LLN metastasis (iLLN) after therapeutic or prophylactic dissection, whereas 28 were recurrent LLN metastasis (rLLN) (Figure 3). All 67 LLN metastases were LLLNs; there were no MLLNs in the current study.

The association between LLN metastasis and clinicopathological features is shown in Table 1. Compared with patients without LLN metastasis (*n* = 878), patients with LLN metastasis (*n* = 67) had high alcohol consumption and advanced clinical N classification (*p* = 0.01 and <0.0001, respectively). The differences in the clinicopathological features between patients with iLLN metastasis and those with rLLN metastasis are also shown in Table 1. rLLN metastasis occurred more frequently in males, those who maintained good oral hygiene, those with an early T-classification, and those with an early N-classification, than iLLN (*p* = 0.04, 0.03, 0.04 and <0.0001, respectively).

### 3.3. Risk Factors for LLN Metastasis

Multivariate logistic regression analysis revealed that habitual alcohol consumption and neck node metastasis were independent risk factors for LLN metastasis (odds ratio 1.93 and 4.58; 95%CI 1.06–3.53 and 2.51–8.35; *p* = 0.032 and < 0.001, respectively) (Table 2). Lingual tumors were more likely to have LLN metastasis than non-lingual tumors, but the difference was not significant (*p* = 0.054).

### 3.4. Prognostic Roles of LLN Metastasis

**Figure 4:**
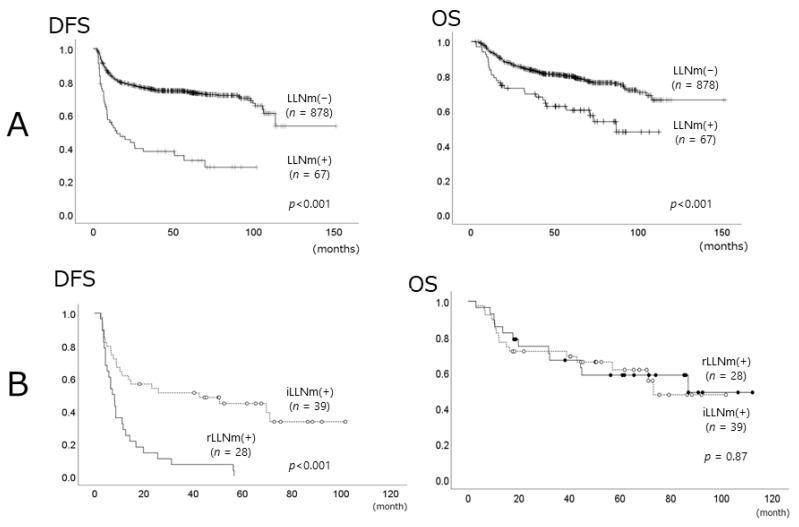
Kaplan–Meier survival curves. (**A**) Comparison of the survival rates (DFS (left panel) and OS (right panel)) of 945 OSCC patients with (*n* = 67) or without LLNm (*n* = 878). (**B**) Comparison of the survival rates (DFS (left panel) and OS (right panel)) of 67 OSCC patients with iLLNm (*n* = 39) or rLLNm (*n* = 28). DFS, disease-free survival; OS, overall survival; OSCC, oral squamous cell carcinoma; LLNm, lingual lymph node metastasis; iLLNm, initial LLN metastasis; rLLNm, recurrent LLN metastasis.

In patients with LLN metastasis, the five-year DFS and OS rates were 32.4% (95% CI: 24.40–24.60%) and 60.3% (95% CI: 47.9–72.6%), respectively. In contrast, the five-year DFS and OS rates of patients without LLN metastasis were 73.5% (95% CI: 70.3–76.6%) and 79.9% (95% CI: 77.1–82.6%), respectively. DFS and OS rates of patients with LLN metastasis were significantly worse than those of patients without LLN metastasis (*p* < 0.001 and *p* < 0.001, respectively) (Figure 4A). Among the patients with LLN metastasis, the median duration of DFS in patients with rLLN was 7.2 months, whereas that of patients with iLLN was 42.5 months. The DFS of patients with rLLN was significantly worse than that of patients with iLLN (*p* < 0.001), but there were no significant differences in overall survival rates between those with iLLN and those with rLLN (Figure 4B). Cox multivariate regression model analysis demonstrated that LLN metastasis was an independent prognostic factor for DFS (HR 3.75, 95% CI 2.53–5.57, *p* < 0.001) and OS (HR 1.95, 95% CI 1.24–3.06, *p* = 0.004) (Table 3).

## 4. Discussion

To the best of our knowledge, this is the first multi-center analysis to evaluate clinicopathological features of patients with LLN metastasis. In this study, 7.1% of patients with OSCC showed LLN metastasis. All LLN metastases were LLLNs, and there were no cases of MLLNs. The tongue was the most frequent primary site (57.1%) of LLN metastasis. Significant risk factors for LLN metastasis included habitual alcohol consumption (OR = 1.93, *p* = 0.032) and clinically determined node-positivity in patients (OR = 4.58, *p* < 0.001). On multivariate analysis, LLN metastasis was shown to be an independent adverse prognostic factor for DFS (HR = 3.75, *p* < 0.001) and OS (HR = 1.95, *p* = 0.004). Prophylactic and/or therapeutic LLLN dissection tended to reduce the disease recurrence rate (*p* = 0.054), but it did not improve the overall survival rate.

LLN is further divided into the subtypes LLLN and MLLN [4]. Metastasis to the MLLN is extremely rare [2,5]. In fact, in the present study, there were no patients with MLLN metastasis. The incidence of LLLN is higher than that of MLLN [5,14,15], but the exact metastasis rate of LLLN remains unknown. Ando et al. [7] reported “para-hyoid node” disease in 6.3% of their 248 patients with early stage oral tongue SCC [7]. Jia et al. reported LLLN swelling in 11 patients (9.9%), three of whom with lingual or oral floor SCC had LLLN metastasis positivity (2.7%) [15]. Fang Q. et al. prospectively investigated the role of LLN in tongue SCC in 231 patients and showed that LLNs were observed in 58 patients (25.1%), 33 of whom had LLN metastasis positivity (14.3%). Of the 33 patients, 28 (12.1%) had isolated LLLN metastasis, three (1.3%) had both MLLN and LLLN, and two (0.8%) had isolated MLLN [14]. The metastatic rates of LLLN varied widely among different studies [7,14,15] (2.7 to 12.1%), which is probably because of differences in the definition of LLLN and the method and/or extent of LLLN dissection.

LLN metastasis usually presents with lingual cancer in an advanced stage [14], but we showed that 24 of 405 patients with non-lingual oral cancer (5.9%) exhibited LLN metastasis. In addition, we demonstrated that even patients with early cT tumors exhibited LLN metastasis (cT1: 4.8% (13 of 273) and cT2: 6.1% (21 of 346)), but the rates were lower than patients with advanced cT tumor (cT3: 9.4% (13 of 139) and cT4: 10.7% (20 of 187)). Note that early stage non-lingual oral cancer can metastasize to LLN.

There were no reliable epidemiological analyses for LLN metastasis. Fang Q. et al. concluded that LLN metastasis was significantly related to lymphovascular invasion, perineural invasion, tumor stage, neck node metastasis, and tumor differentiation with univariate analysis [14]. It was further demonstrated that higher pretreatment NLR was significantly correlated with LLN metastasis, and Lin et al. mentioned that high NLR may indicate LLN dissection in patients with early tongue cancer [16]. In this study, however, pretreatment NLR was not significantly different between LLN-positive and LLN-negative patients. Pretreatment NLR may be a non-specific parameter because it could be influenced by concomitant conditions, such as infections or inflammation [17]. In the current study, multivariate analysis revealed clinical neck node metastasis as an independent risk factor of LLN metastasis, which corresponded with previous reports [14,15]. In addition, habitual alcohol consumption was also revealed as an independent risk factor. Alcohol consumption can not only enhance carcinogenesis, but also increase the aggressiveness and malignancy of existing tumors [18]. In a cross-sectional observation study of the pathological features in patients with head and neck cancers (*n* = 1633) according to the smoking and drinking habits, alcohol consumption was related to nodal metastasis, whereas smoking correlated with the degree of differentiation [19]. Therefore, we may consider the possibility of LLN metastasis and alter the management of LLN if we see node-positive patients reporting habitual alcohol consumption.

The prognostic role of LLN metastasis is not well known. Fang Q. et al. reported that the five-year locoregional control rate in patients with LLN metastasis was 45%. This was significantly worse than the 65% five-year locoregional control rate of patients without LLN metastasis (*p* = 0.013), which was confirmed by multivariate analysis. In a recently published article, Yang W. et al. analyzed 317 patients with early stage tongue SCC (cT1-2N0) using multivariate analysis, and showed that the locoregional control rates and disease-specific survival rates of patients with LLN metastasis were significantly worse than in patients without LLN (HR: 1.999 and 1.845; *p* = 0.015 and <0.001, respectively). In our study, LLN metastasis was found to be an independent adverse prognostic factor for DFS (HR = 3.75, *p* < 0.001) and OS (HR = 1.95, *p* = 0.004). However, LLN dissection did not improve OS outcomes, although LLN dissection tended to improve DFS (the results did not reach statistical significance, *p* = 0.054).

This study has some limitations. Firstly, this was a retrospective multicenter study assessing heterogeneous data. Thus, variability in the surgeons’ skills and therapeutic modality may have affected the results. Secondly, in the current study, those cases with missing data were deleted and the remaining data were analyzed. This may have introduced bias in the estimation of the parameters. Thirdly, the percentage of LLN metastases (7.1%) was relatively low, which might have resulted in a lack of statistical power to evaluate the influence of LLN dissection on the improvement of DFS. Although the current results did not reach statistical significance (*p* = 0.054), we recommend that LLN dissection should be routinely performed, especially for patients at high risk of LLN metastasis (habitual alcohol consumers and/or patients with clinical neck node metastasis), because this procedure can be safely performed with a low risk of postoperative sequelae. Finally, there are no gold standard indications and/or methods for LLN dissection. Theoretically, if we do not perform total glossectomy, we cannot remove occult LLN metastasis completely. This indicates that the extent of LLN dissection in the current study might be insufficient, especially for patients who underwent partial glossectomy using an exclusively transoral approach. Therefore, further prospective studies are required to accurately assess which OSCC patients should undergo LLN dissection and to determine the extent to which the procedure could be performed in order to improve survival outcomes.

## 5. Conclusions

LLN metastasis was noted in 67 patients (7.1%) out of 945 patients with OSCC. All LLN metastases were lateral LLN, and there were no cases of median LLN. Habitual alcohol consumption and clinical neck node metastasis were independent risk factors for LLN metastasis. LLN metastasis was an independent negative prognostic factor for disease-free and overall survival, but LLN dissection did not improve survival outcomes. The study population (relatively low percentage of LLN metastases) may have resulted in a lack of statistical power. Therefore, further prospective study in a larger cohort of these relatively rare LLN metastatic patients through a multicenter collaboration is needed to fully assess the indication and the extent of LLN dissection required in OSCC patients.

## Figures and Tables

**Table 1:** Clinical characteristics of patients.

Variables		Overall *n* = 945	Overall *n* = 945			LLN Metastasis (+) *n* = 67		
			LLN Metastasis (−) *n* = 878 (92.9%)	LLN Metastasis (+) *n* = 67 (7.1%)	*p*-Value	Initial LLN Metastasis *n* = 39 (58.2%)	Recurrent LLN Metastasis *n* = 28 (41.8%)	*p*-Value
Clinical backgrounds								
Age								
	>70, *n* (%)	436 (46.1)	409 (46.6)	27 (40.3)	0.37	12 (30.8)	13 (46.4)	0.19
	≤70, *n* (%)	509 (53.9)	469 (53.4)	40 (59.7)		27 (69.2)	15 (53.4)	
Sex								
	Male, *n* (%)	580 (61.3)	534 (60.8)	46 (68.7)	0.24	23 (59.0)	23 (82.1)	0.04 *
	Female, *n* (%)	365 (38.7)	344 (39.2)	21 (31.3)		16 (41.0)	5 (17.9)	
Subsite								
	Lingual, *n* (%)	540 (57.1)	497 (56.6)	43 (64.2)	0.25	23 (59.0)	20 (71.4)	0.29
	Non-lingual, *n* (%)	405 (42.9)	381 (43.4)	24 (35.8)		16 (41.0)	8 (28.6)	
	Gingiva, *n* (%)	208 (22.0)	198 (22.6)	10 (14.9)		7 (17.9)	3 (10.7)	
	Oral floor, *n* (%)	99 (10.5)	90 (10.3)	9 (13.4)		7 (17.9)	2 (7.1)	
	Buccal, *n* (%)	79 (8.4)	75 (8.5)	4 (6.0)		2 (5.1)	2 (7.1)	
	Hard palate, *n* (%)	13 (1.4)	12 (1.4)	1 (1.5)		0 (0)	1 (3.6)	
	Lip, *n* (%)	6 (0.6)	6 (0.7)	0 (0)		0 (0)	0 (0)	
Tumor location								
	Not beyond midline, *n* (%)	813 (86.0)	760 (86.6)	53 (79.1)	0.10	27 (69.2)	26 (92.9)	0.02 *
	Beyond midline, *n* (%)	132 (14.0)	118 (13.4)	14 (20.9)		12 (30.8)	2 (7.1)	
Alcohol								
	None/Sometimes, *n* (%)	514 (54.4)	488 (55.6)	26 (38.8)	0.01 *	16 (41.0)	10 (35.7)	0.66
	Habitual, *n* (%)	431 (45.6)	390 (44.4)	41 (61.2)		23 (59.0)	18 (64.3)	
Smoking								
	Never/Former, *n* (%)	699 (74.0)	651 (74.1)	48 (71.6)	0.67	28 (71.8)	20 (71.4)	0.97
	Current, *n* (%)	246 (26.0)	227 (25.9)	19 (28.4)		11 (28.2)	8 (28.6)	
Oral hygiene								
	Good, *n* (%)	631 (66.8)	591 (67.3)	40 (59.7)	0.23	19 (48.7)	21 (75.0)	0.03 *
	Poor, *n* (%)	314 (33.2)	287 (32.7)	27 (40.3)		20 (51.3)	7 (25.0)	
DM								
	−, *n* (%)	807 (85.4)	746 (85.0)	61 (91.0)	0.21	34 (87.2)	27 (96.4)	0.19
	+, *n* (%)	138 (14.6)	132 (15.0)	6 (9.0)		5 (12.8)	1 (3.6)	
Initial clinical/laboratory findings								
cT classification								
	T1, *n* (%)	273 (28.9)	260 (29.6)	13 (19.4)	0.06	4 (10.3)	9 (32.1)	0.04 *
	T2, *n* (%)	346 (36.6)	325 (37.0)	21 (31.3)		11 (28.2)	10 (35.7)	
	T3, *n* (%)	139 (14.7)	126 (14.4)	13 (19.4)		8 (20.5)	5 (17.9)	
	T4, *n* (%)	187 (19.8)	167 (19.0)	20 (29.9)		16 (41.0)	4 (14.3)	
cN classification								
	N0, *n* (%)	646 (68.4)	622 (70.8)	24 (35.8)	<0.0001 *	4 (10.3)	20 (71.4)	<0.0001 *
	N1, *n* (%)	117 (12.4)	104 (11.8)	13 (19.4)		9 (23.1)	4 (14.3)	
	N2, *n* (%)	169 (17.9)	143 (16.3)	26 (38.8)		22 (56.4)	4 (14.3)	
	N3, *n* (%)	13 (1.4)	9 (1.0)	4 (6.0)		4 (10.3)	0 (0)	
NLR								
	Median	2.3	2.3	2.45	0.75	2.7	2.2	0.26
	Range	0.4–39.6	0.4–39.6	0.6–8.4		1.0–5.3	0.6–8.4	
Histological findings								
Tumor differentiation								
	Well or Moderate, *n* (%)	892 (94.4)	828 (94.3)	64 (95.5)	0.68	37 (94.9)	27 (96.4)	0.76
	Poor, *n* (%)	53 (5.6)	50 (5.7)	3 (4.5)		2 (5.1)	1 (3.6)	

LLN: lingual lymph node, DM: diabetes mellitus, NLR: neutrophil−lymphocyte ratio. * statistically significant values.

**Table 2:** Multivariate analyses for prediction of LLN metastasis.

				Adjusted OR		
				OR	95%CI	*p*-Value
Clinical backgrounds						
Age	>70	vs.	≤70	0.92	0.53–1.61	0.771
Sex	Male	vs.	Female	1.02	0.55–1.90	0.947
Subsite	Lingual	vs.	Non-lingual	1.74	0.99–3.05	0.054
Tumor location	Beyond midline	vs.	Not beyond midline	1.29	0.64–2.56	0.476
Alcohol	habitual	vs.	none/sometimes	1.93	1.06–3.53	0.032 *
Smoking	current	vs.	never/former	0.73	0.40–1.36	0.323
Oral hygiene	Poor	vs.	Good	1.36	0.79–2.27	0.284
DM	+	vs.	−	0.50	0.21–1.22	0.128
Initial clinical/laboratory findings						
cT classification	T3/4	vs.	T1/2	0.99	0.53–1.82	0.961
cN classification	+	vs.	−	4.58	2.51–8.35	<0.001 *
NLR	high	vs.	low	1.14	0.67–1.93	0.636
Histological findings						
Tumor differentiation	Poor	vs.	Well or Moderate	0.61	0.18–2.07	0.425

DM: diabetes mellitus, NLR: neutrophil−lymphocyte ratio, OR: odds ratio. * statistically significant values.

**Table 3:** Multivariate analysis of clinicopathological risk parameters on survival outcomes.

Covariate				Disease−Free Survival			Overall Survival		
				AdjustedHR	95%CI	*p* Value	AdjustedHR	95%CI	*p* Value
Clinical backgrounds									
Age	>70	vs.	≤70	1.28	0.98–1.67	0.073	1.41	1.03–1.91	0.03 *
Sex	Male	vs.	Female	0.85	0.65–1.12	0.258	1.40	1.00–1.96	0.048 *
Subsite	Lingual	vs.	Non-lingual	1.04	0.80–1.35	0.768	0.87	0.64–1.17	0.342
Tumor location	Beyond midline	vs.	Not beyond midline	1.14	0.80–1.62	0.485	1.39	0.97–1.99	0.076
Alcohol	habitual	vs.	none/sometimes	1.05	0.80–1.39	0.722	0.79	0.57–1.08	0.136
Smoking	current	vs.	never/former	1.13	0.83–1.50	0.477	1.52	1.09–2.12	0.013 *
Oral hygiene	Poor	vs.	Good	1.02	0.79–1.32	0.864	0.92	0.68–1.24	0.569
DM	+	vs.	−	1.00	0.70–1.43	0.989	1.21	0.83–1.77	0.320
Initial clinical/laboratory findings									
cT classification	T3/4	vs.	T1/2	1.60	1.15–2.23	0.005 *	1.79	1.23–2.59	0.002 *
cN classification	+	vs.	−	0.87	0.62–1.23	0.429	1.25	0.85–1.82	0.255
NLR	high	vs.	low	0.97	0.76–1.25	0.825	1.26	0.95–1.68	0.113
Treatment									
NAC	Not performed	vs.	Performed	0.67	0.50–0.89	0.006 *	0.92	0.66–1.28	0.620
Primary resection	Transoral approach	vs.	Pull-through resection	0.89	0.63–1.26	0.514	1.05	0.73–1.50	0.813
Neck dissection	Not performed	vs.	Performed	1.18	0.80–1.73	0.400	0.83	0.53–1.31	0.434
PORT	Not performed	vs.	Performed	0.79	0.54–1.14	0.206	0.83	0.55–1.24	0.356
Histological findings									
Resection margin	Positive	vs.	Negative	1.79	1.23–2.62	0.002 *	1.73	1.12–2.68	0.014 *
ECE	+	vs.	−	1.70	1.06–2.73	0.029 *	1.96	1.21–3.18	0.006 *
Tumor differentiation	Poor	vs.	Well or Moderate	1.91	1.26–2.88	0.002 *	2.12	1.37–3.28	0.001 *
LLN									
LLN dissection	Not performed	vs.	Performed	1.41	0.99–1.99	0.054	1.27	0.88–1.81	0.197
LLN metastasis	+	vs.	−	3.75	2.53–5.57	<0.001 *	1.95	1.24–3.06	0.004 *

DM: diabetes mellitus, NLR: neutrophil−lymphocyte ratio, NAC: neoadjuvant chemotherapy, PORT: postoperative radiotherapy, ECE: extra-capsular extension, LLN: lingual lymph node. * statistically significant values.

## Data Availability

The data presented in this study are available on request from the corresponding author.

## References

[1] Werner J.A., Dünne A.A., Myers J.N. (2003). Functional anatomy of the lymphatic drainage system of the upper aerodigestive tract and its role in metastasis of squamous cell carcinoma. Head Neck.

[2] Ozeki S., Tashiro H., Okamoto M., Matsushima T. (1985). Metastasis to the lingual lymph node in carcinoma of the tongue. J. Maxillofac. Surg..

[3] Woolgar J.A. (1999). Histological distribution of cervical lymph node metastases from intraoral/oropharyngeal squamous cell car-cinomas. Br. J. Oral Maxillofac. Surg..

[4] Rouvière H. (1938). Anatomy of the Human Lymphatic System: A Compendium Translated from the Original ‘Anatomie des Lymphatiques de l’Homme’and Rearranged for the Use of Students and Practitioners by MJ Tobias.

[5] Ananian S.G., Gvetadze S.R., Ilkaev K.D., Mochalnikova V.V., Zayratiants G.O., Mkhitarov V.A., Yang X., Ciciashvili A.M. (2015). Anatomic-histologic study of the floor of the mouth: The lingual lymph nodes. Jpn. J. Clin. Oncol..

[6] Eguchi K., Muro S., Miwa K., Yamaguchi K., Akita K. (2020). Deep cervical fascia as an anatomical landmark of lingual lymph nodes: An anatomic and histologic study. Auris Nasus Larynx.

[7] Ando M., Asai M., Asakage T., Oyama W., Saikawa M., Yamazaki M., Miyazaki M., Ugumori T., Daiko H., Hayashi R. (2009). Metastatic neck disease beyond the limits of a neck dissection: Attention to the ’para-hyoid’ area in T1/2 oral tongue cancer. Jpn. J. Clin. Oncol..

[8] Ando M., Asai M., Ono T., Nakanishi Y., Asakage T., Yamasoba T. (2010). Metastases to the lingual nodes in tongue cancer: A pitfall in a conventional neck dissection. Auris Nasus Larynx.

[9] Umeda M., Minamikawa T., Shigeta T., Oguni A., Kataoka T., Takahashi H., Shibuya Y., Komori T. (2010). Metastasis to the lingual lymph node in patients with squamous cell carcinoma of the floor of the mouth: A report of two cases. Kobe J. Med. Sci..

[10] Saito M., Nishiyama H., Oda Y., Shingaki S., Hayashi T. (2012). The lingual lymph node identified as a sentinel node on CT lympho-graphy in a patient with cN0 squamous cell carcinoma of the tongue. Dentomaxillofac. Radiol..

[11] Dinardo L.J. (1998). Lymphatics of the Submandibular Space: An anatomic, clinical, and pathologic study with applications to floor-of-mouth carcinoma. Laryngoscope.

[12] Dutton J.M., Graham S.M., Hoffman H.T. (2002). Metastatic cancer to the floor of mouth: The lingual lymph nodes. Head Neck.

[13] Eguchi K., Kawai S., Mukai M., Nagashima H., Shirakura S., Sugimoto T., Asakage T. (2020). Medial lingual lymph node metastasis in carcinoma of the tongue. Auris Nasus Larynx.

[14] Fang Q., Li P., Qi J., Luo R., Chen D., Zhang X. (2019). Value of lingual lymph node metastasis in patients with squamous cell carcinoma of the tongue. Laryngoscope.

[15] Jia J., Jia M.-Q., Zou H.-X. (2018). Lingual lymph nodes in patients with squamous cell carcinoma of the tongue and the floor of the mouth. Head Neck.

[16] Lin W.-J., Wang C.-C., Chen S.-H. (2019). In reference to Value of lingual lymph node metastasis in patients with squamous cell carcinoma of the tongue. Laryngoscope.

[17] Yang W., Sun M., Jie Q., Zhou H., Zhang P., Zhu J. (2020). Lingual Lymph Node Metastasis in cT1-2N0 Tongue Squamous Cell Carcinoma: Is It an Indicator for Elective Neck Dissection. Front. Oncol..

[18] Xu M., Luo J. (2017). Alcohol and Cancer Stem Cells. Cancers.

[19] Moyses R., Lopez R., Cury P., Siqueira S., Curioni O., Filho J.G., Figueiredo D., Tajara E., Michaluart P. (2013). Significant differences in demographic, clinical, and pathological features in relation to smoking and alcohol consumption among 1633 head and neck cancer patients. Clinics.

